# Relevance of Morning and Evening Energy and Macronutrient Intake during Childhood for Body Composition in Early Adolescence

**DOI:** 10.3390/nu8110716

**Published:** 2016-11-10

**Authors:** Tanja Diederichs, Sarah Roßbach, Christian Herder, Ute Alexy, Anette E. Buyken

**Affiliations:** 1 IEL-Nutritional Epidemiology, DONALD Study, University of Bonn, Heinstueck 11, 44225 Dortmund, Germany; tdiederi@uni-bonn.de (T.D.); srossbac@uni-bonn.de (S.R.); alexy@uni-bonn.de (U.A.); 2 Institute for Clinical Diabetology, German Diabetes Center, Leibniz Center for Diabetes Research at Heinrich Heine University Düsseldorf, Auf’m Hennekamp 65, 40225 Düsseldorf, Germany; Christian.Herder@ddz.uni-duesseldorf.de; 3 German Center for Diabetes Research (DZD), Ingolstädter Landstr. 1, 85764 München-Neuherberg, Germany

**Keywords:** childhood, adolescence, morning intake, evening intake, macronutrient intake, fat mass

## Abstract

(1) Background: This study investigated the relevance of morning and evening energy and macronutrient intake during childhood for body composition in early adolescence; (2) Methods: Analyses were based on data from 372 DONALD (DOrtmund Nutritional and Anthropometric Longitudinally Designed study) participants. Explorative life-course plots were performed to examine whether morning or evening energy and macronutrient intake at 3/4 years, 5/6 years, or 7/8 years is critical for fat mass index (FMI [kg/m^2^]) and fat free mass index (FFMI [kg/m^2^]) in early adolescence (10/11 years). Subsequently, exposures in periods identified as consistently critical were examined in depth using adjusted regression models; (3) Results: Life-course plots identified morning fat and carbohydrate (CHO) intake at 3/4 years and 7/8 years as well as changes in these intakes between 3/4 years and 7/8 years as potentially critical for FMI at 10/11 years. Adjusted regression models corroborated higher FMI values at 10/11 years among those who had consumed less fat (*p* = 0.01) and more CHO (*p* = 0.01) in the morning at 7/8 years as well as among those who had decreased their morning fat intake (*p* = 0.02) and increased their morning CHO intake (*p* = 0.05) between 3/4 years and 7/8 years; (4) Conclusion: During childhood, adherence to a low fat, high CHO intake in the morning may have unfavorable consequences for FMI in early adolescence.

## 1. Introduction

Primary school years have recently been identified as a potentially “critical period” for the development and persistence of overweight and obesity [1] in different Western societies [2,3]. Moreover, children with a body mass index (BMI) in the upper normal range before and during primary school were at higher risk for obesity development until the end of primary school compared to those who had a BMI in the lower range [4]. These developments may in part be attributable to the considerable changes in children’s daily routine, as entry into institutions like kindergarten and primary school results in an externally determined time window for morning intake and affects the timing and duration of evening intake due to less flexible bed times. These changes in circadian (=approximately 24 h) rhythmicity may entail changes in morning and evening energy and macronutrient intake.

Among adults, severe disruptions of circadian rhythm induced by shiftwork or jetlag are known to increase the risk for obesity, type 2 diabetes, and cardiovascular diseases [5,6]. Similar associations have been suggested for moderate disruptions due to irregular meal times or delayed bed times [7]. Moreover, studies among adults revealed that a number of metabolic processes follow circadian rhythms: levels of hunger are known to increase over the day [8], accompanied by a reduced ability to compensate for higher evening energy intakes [9]; in addition, insulin sensitivity [10] and diet-induced thermogenesis [11] may decrease over the day. It is hence plausible that day-time specific energy or macronutrient intake—in particular, if not in balance with the circadianity of these metabolic processes—may have longer-term effects on body composition. However, evidence of these aspects among children is lacking. This study therefore examined, whether morning or evening energy and macronutrient intake during kindergarten and primary school age is of prospective relevance for body composition in early adolescence (i.e., the end of primary school). 

## 2. Methods

### 2.1. DONALD Study

The DONALD (DOrtmund Nutritional and Anthropometric Longitudinally Designed) study is an ongoing open cohort study conducted in Dortmund, Germany, which was previously described in detail [9]. Briefly, since recruitment began in 1985, detailed information on diet, growth, development, and metabolism between infancy and early adulthood has been collected from >1550 children. Every year, 35 to 40 healthy infants with no prevalent diseases affecting growth and/or diet are newly recruited and first examined at the ages of three or six months. Each child returns for three more visits in the first year, two in the second, and then once annually until early adulthood. The health status is re-assessed at each visit.

The study was approved by the Ethics Committee of the University of Bonn; all examinations are performed with parental and later the children’s written consent.

### 2.2. Nutritional Data

Nutritional data are assessed by 3-day weighed dietary records on three consecutive days. Participants are free to choose the days of recording. Overall, 37% of the 3–8 years old participants eligible for the present analysis chose to record their intake over three consecutive weekdays only, while 19% documented their intake on two weekdays and one weekend day, and 44% chose one weekday and two weekend days. Parents and/or participants are instructed by dietitians to weigh all foods and beverages consumed by the participant, including leftovers, to the nearest 1 g over three consecutive days with the use of regularly calibrated electronic food scales (initially Soehnle Digita 8000 (Leifheit AG, Nassau, Germany), now WEDO digi 2000 (Werner Dorsch GmbH, Muenster/Dieburg, Germany)). Semi-quantitative measures (e.g., number of spoons) are allowed when exact weighing is not possible. Information on recipes and on the types and brands of food items consumed is also requested. The dietary records are analyzed using the continuously updated in-house nutrient database LEBTAB [10], which includes information from standard nutrient tables, product labels, or recipe simulations based on the listed ingredients and nutrients. Additionally, the time of every eating occasion is recorded.

### 2.3. Anthropometric Data

Participants are measured at each visit by trained nurses according to standard procedures (WHO 1995), dressed in underwear only and barefoot. From the age of two onward, standing height is measured to the nearest 0.1 cm with a digital stadiometer. Weight is measured to the nearest 0.1 kg with an electronic scale (model 753 E; Seca, Hamburg, Germany). Skinfold thicknesses are measured from the age of six months onward on the right side of the body at the biceps, triceps, subscapular, and suprailiac sites to the nearest 0.1 mm with a Holtain caliper (Holtain Ltd., Crymych, UK). For assurance of quality data, inter- and intra-observer agreement is monitored regularly (average inter- and intra-individual variation coefficients obtained between 2005 and 2015 were 10.0% and 13.0% for biceps, 4.5% and 5.7% for triceps, 5.0% and 7.6% for subscapular, and 7.6% and 9.0% for suprailiacal skinfolds).

Body mass index (BMI, kg/m^2^) was calculated, percent body fat (%BF) was estimated for children up to age 8 years from all four skinfolds using the Deurenberg equation [11], and for children above age 8 years, from two skinfolds (triceps, subscapular) using the Slaughter equation [12]. Body fat mass (kg) and fat-free body mass (kg) were calculated (“(%BF × body mass)/100” and “((100 − %BF) × body mass)/100”, respectively) and related to the square of height to obtain fat mass index (FMI, kg/m^2^) and fat free mass index (FFMI, kg/m^2^). Since the distribution of FMI was skewed, log-transformed values were used in analyses.

### 2.4. Familial Characteristics

On their child’s admission to the study and at regular intervals thereafter, parents are interviewed concerning the child’s early life data as well as familial and socio-economic characteristics. Additionally, parents are weighed and measured. Information on birth anthropometrics and gestational age are abstracted from a standardized document (Mutterpass) given to all pregnant women in Germany.

### 2.5. Definition of Morning and Evening

For estimation of morning and evening energy and macronutrient intake, food consumption documented between an age-specific end of the night and 11 a.m. and between 6 p.m. and an age-specific start of the night was used. The cut-points at 11 a.m. in the morning and 6 p.m. in the evening emerged in a preliminary analysis of DONALD data, showing that children and adolescents (2–18 years) consume their first (and second) breakfast until 11 a.m., and that 6 p.m. marks the point in time between afternoon snacks and the main evening meals. The age-specific start and end of the night was estimated using all available consecutive 3-day dietary records from DONALD participants of the respective age. The age-specific start of the night was defined as the time of the day, after which less than 5% of the last eating occasions (≥10 kcal) before midnight were documented; the age-specific end of the night was the time of the day past 5 a.m., after which more than 5% of the first eating occasions (≥10 kcal) were documented.

### 2.6. Study Sample

The present analysis included term (gestational age 37–42 weeks) singletons with a minimum birth weight of 2500 g. Regarding exposure data, weighed dietary records had to be available from three consecutive days to allow the derivation of the start and end of the night. The outcomes FMI and FFMI were estimated from the latest available anthropometric measurement at age 10 or 11 years.

In an initial explorative analysis, we were interested in the identification of one or more potentially critical time period(s) for later body composition. To this end, life-course plots (see statistical analysis) allow a first data screening. For this analysis, we considered participants who had provided at least one 3-day weighed dietary record in each of the three potentially critical time periods 3/4 years, 5/6 years, and 7/8 years, and anthropometric data at age 10/11 years (*N* = 499). Additionally, data on the age marking the start of the pubertal growth spurt “age at take-off” (ATO) had to be available, resulting in a final sample size of *N* = 372 for the explorative analysis. 

For the in-depth analyses, more strict inclusion criteria were used. We only considered participants who had provided two plausible 3-day weighed dietary records and additional covariates (see statistical analysis) for the respective time periods identified by the explorative analysis. A 3-day weighed dietary record was considered plausible when the total recorded energy intake was adequate in relation to the estimated basal metabolic rate (BMR) using modified age-dependent cutoffs from Goldberg et al. [13].

### 2.7. Statistical Analysis

SAS procedures (SAS version 9.2, SAS Institute, Cary, NC, USA) were used for data analysis. Significance level was set at *p* < 0.05; *p* < 0.1 was considered to indicate a trend. Since there were no interactions between sex and the exposure–outcome associations, data from boys and girls were pooled. The statistical analysis was performed in a two-stage approach, starting with an initial explorative analysis followed by an in-depth analysis.

For the initial explorative analysis, life-course plots [14] were used to simultaneously consider a repeatedly measured exposure (e.g., morning fat intake at 3/4 years, 5/6 years, 7/8 years) in relation to one outcome (e.g., FMI at 10/11 years). Energy intake in the morning and in the evening was expressed as percentage of total daily energy intake (TEI); morning and evening macronutrient intake was expressed as percentages of fat, carbohydrate (CHO), or protein of energy intake before 11 a.m. and after 6 p.m., respectively. For all three potentially critical periods (i.e., 3/4 years, 5/6 years, 7/8 years), exposures were averaged (if two records were available), checked for normal distribution, transformed (if necessary), and standardized (mean = 0, standard deviation = 1) by critical time period. Multivariable linear regression models were calculated with continuous macronutrient intake as independent exposures and FMI or FFMI as outcomes (adjusted for ATO and mean standardized TEI between age 3–8 years). The resulting regression coefficients were plotted against age, and both their own value (representing the strength of the relation at a distinct time point) and their changes (representing the association between outcome and the exposure’s change over time) and were evaluated to identify a potentially critical time period of one or more intake variables with respect to body composition in early adolescence.

The in-depth multivariable linear regression analyses focused on the time period(s) and specific intake (morning/evening, energy/macronutrient) identified by the life-course plot approach. Energy-adjusted morning and evening macronutrient intakes were calculated using the residual method [15]. Intakes were entered individually as independent exposures together with ATO into multivariable linear regression models (crude models). Predicted means of FMI or FFMI were presented by tertiles of the respective intake. In a further step, potentially confounding factors were considered as covariates: sex (male/female), FMI- or FFMI at the analysis-specific baseline (i.e., 3/4 years, 5/6 years, or 7/8 years), birth year, appropriateness for gestational age (i.e., whether birth weight and length were appropriate for gestational age yes/no), full breastfeeding (≥4 months yes/no), maternal overweight (≥25 kg/m^2^ yes/no), maternal educational status (≥12 years of schooling yes/no) and smoking in the household (yes/no). Only covariates which modified the exposure’s regression coefficient in the crude model by ≥10% [16] or were independent predictors of the outcome variable [17] were included into a hierarchical approach of covariate selection [18]. The following hierarchy was used: (1) general characteristics (sex and baseline FMI- or FFMI); (2) early life characteristics (birth year, appropriateness for gestational age, full breastfeeding); (3) familial and socio-economic characteristics (maternal overweight, maternal educational status, smoking in the household). To ensure comparability, model building was performed for the strongest exposure–outcome association per time period and outcome. Identified confounders were also used in all other models. Sensitivity analyses using individually constructed models for each exposure–outcome association yielded similar results. Additional sensitivity analyses were performed to address the possibility that observed prospective relations were driven by cross-sectional associations of the respective predictor at age 10/11 years with body composition at age 10/11 years.

## 3. Results

### 3.1. Explorative Analysis

Table 1 gives the characteristics of the explorative life-course plot analysis sample (*N* = 372). According to the cut-points of the International Obesity Task Force (IOTF) [19], 16% of the children were overweight at 10/11 years; according to McCarthy [20], 21% had an excessive body fatness.

Nutritional data of the explorative sample are presented in Table 2 for each potentially critical time period. Morning, evening, and daily fat consumption decreased with age, whereas CHO consumption increased; protein intake was broadly consistent. Regardless of age, children consumed more CHO and less fat or protein in the morning compared to the evening. 

**Figure 1:**
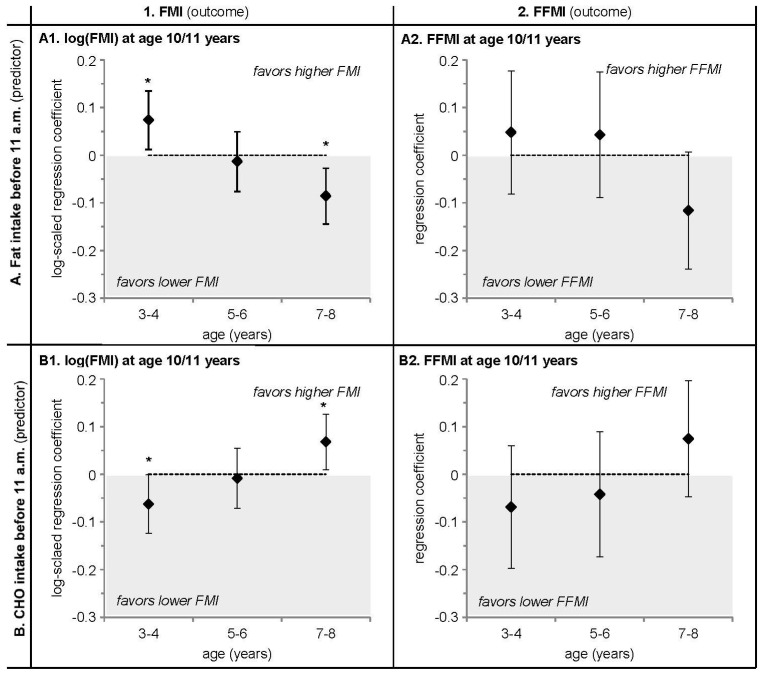
Life-course plots of multivariable linear regression analyses with log-transformed FMI (kg/m^2^) and crude FFMI (kg/m^2^) in early adolescence at 10/11 years as the outcome and the standardized intake of (**A**) morning fat (% of energy) and (**B**) morning carbohydrate (CHO) (% of energy) as predicting variables. Analyses were adjusted for age at take-off (ATO) and mean standardized daily energy intake (TEI) from 3 to 8 y. Values are regression coefficients (95% CI) from models ran on *N* = 372 participants of the DONALD study. * *p* < 0.05. ATO—age at take-off, CHO—carbohydrates, FFMI—fat free mass index (kg/m^2^), FMI—fat mass index (kg/m^2^).

The explorative life-course plot analysis (adjusted for ATO and TEI) consistently revealed associations of morning fat and morning CHO intake with FMI at 10/11 years (Figure 1A1,B1). Similar, albeit non-significant, associations were seen with FFMI (Figure 1A2,B2). Life-course plots show that a higher fat intake at 3/4 years was related to a higher FMI at 10/11 years (*p* = 0.02) (A1), just as a lower CHO intake was related to a higher FMI (*p* = 0.04) (B1). These trends reversed at 7/8 years (%fat *p* = 0.004, %CHO *p* = 0.03 before 11 a.m.), when a higher fat intake (A1) and a lower CHO intake (B1) before 11 a.m. were related to a lower FMI at 10/11 years. Moreover, the clear switch in signs of the regression coefficients between 3/4 years and 7/8 years suggested an additional relevance of the change in macronutrient intake over this time course (A1, B1) [21]. Morning energy or protein and evening energy or macronutrient intakes at 3/4 years, 5/6 years, or 7/8 years were not associated with FMI or FFMI at 10/11 years (data not shown).

### 3.2. In-Depth Analysis

On the basis of the results of the life-course plots, subsequent in-depth regression analyses (adjusted for ATO and baseline FMI or FFMI, respectively) focused on the prospective relevance of morning fat and CHO intake at 3/4 years and 7/8 years, as well as the change in these intakes between 3/4 years and 7/8 years for FMI at 10/11 years. Characteristics of the in-depth analyses sample (*N* = 297) were very similar to the explorative analysis sample (Supplementary Material Tables S1 and S2).

In these in-depth analyses, morning %CHO and %fat intake at age 3/4 years were not relevant for FMI at age 10/11 years (%fat *p* = 0.93, %CHO *p* = 0.80) (Table 3). By contrast, higher morning %fat intake and lower morning %CHO intake at 7/8 years continued to be predictive of a lower FMI at 10/11 years in multivariable models (%fat *p* = 0.01, %CHO *p* = 0.01). Moreover, a decrease in morning %fat intake and an increase in morning %CHO intake between 3/4 years and 7/8 years was related to a higher FMI at 10/11 years (Δ%fat *p* = 0.02, Δ%CHO *p* = 0.05). Additional adjustment for the corresponding macronutrient intake at age 10/11 years did not change the associations of morning %CHO or %fat intake at age 7/8 years or Δ%fat or Δ%CHO with FMI at 10/11 years (data not shown). For comparative purposes, we also ran multivariable regression models with FFMI as an outcome (Supplementary Material Table S3). However, neither morning %fat nor morning %CHO intake at 3/4 years or 7/8 years nor the change in intakes between 3/4 years and 7/8 years were related to FFMI at 10/11 years. 

## 4. Discussion

The present study provides first evidence for a prospective relevance of children’s habitual macronutrient intake on a day-time specific level for their fat mass in early adolescence. Our results indicate that a higher FMI in early adolescence (i.e., around the end of primary school) may result if primary school children have a habitual low fat, high CHO intake during the morning, and if children decrease their morning fat intake and increase their morning CHO intake from kindergarten to primary school age. 

For the interpretation of our results it is important to note that the analyzed data were collected in healthy free-living children following their habitual dietary habits. Morning CHO intake was moderate to high (age-dependent tertile medians: 44E% to 60E%, Table 3), and morning fat intake was low to moderate (age-dependent tertile medians: 27E% to 42E%, Table 3). Examples for low fat, high CHO breakfasts were ready-to-eat cereal with milk or bread with jam, often combined with juice and/or fruits and/or sweetened milk beverages. In turn, examples of moderate-fat, low-CHO breakfasts were oat(-nuts)-cereal with milk, bread with cheese, or sausage/ham or eggs, often combined with fruits or vegetables. Hence, modest differences in food choices translated into considerable differences in morning macronutrient intakes and subsequent fat mass in early adolescence.

Mechanisms to explain our results are unknown, but the following possible reasons are plausible and should be considered. 

Breakfasts with a lower CHO-to-fat ratio compared to a higher one elicit lower overall postprandial blood glucose and insulin responses [22]. This may have consequences for levels of hunger/satiety and subsequent energy intake. In 64 overweight adults, a low CHO, high fat breakfast compared to a high CHO, low fat breakfast resulted in lower levels of hunger and a higher perceived fullness [22]. Data from children and adolescents stem largely from studies analyzing breakfast glycemic index (GI), supporting a short-term role of breakfast GI for subsequent levels of hunger and energy intake at lunch, as a low-GI compared to a high-GI breakfast resulted in lower levels of hunger and energy intake at lunch among normal weight or obese children and adolescents [23,24]. Beyond these acute effect studies, there are no intervention studies analyzing the longer-term impact of modulating breakfast fat/CHO content on body composition development among children. Of note, in our study, additional analyses did not confirm a role of morning dietary GI in the prospective associations of morning carbohydrate intake with adolescent body composition (data not shown). Nonetheless, higher postprandial blood glucose and insulin responses elicited by the high morning carbohydrate intake may be a relevant mechanism for our observation.

The specificity of our findings for morning intakes may also reflect a relevance of circadianity. Postprandial insulin and blood glucose responses follow a diurnal rhythm, at least in adults. Two small studies suggest a decrease in insulin sensitivity throughout the day [25,26], which would imply that a high morning CHO intake would be preferable over a high evening CHO intake. In fact, CHO intake was 6E%–7E% (absolute) higher in the morning than in the evening in our sample (Table 2). However, for our observation of an unfavorable association between higher morning CHO intakes and subsequent fat mass, two physiologically occurring phenomena may play a decisive role. First, pubertal insulin resistance, characterized by a decrease in peripheral insulin sensitivity accompanied by a lower increase in acute insulin response [27], emerges as early as at age 7 years [28]. This may translate into an enhanced vulnerability to high CHO loads among primary school children. Second, the dawn phenomenon, defined as an early morning rise in blood glucose levels and/or insulin requirements without food intake, was observed in patients with diabetes mellitus [29], as well as in healthy individuals [30]. Data for healthy children and adolescents on the dawn phenomenon are scarce and controversial: a difference in insulin levels during the night was neither observed in 31 healthy children and adolescents [31] nor in 10 healthy adolescents [32]. However, for the latter, a significantly higher insulin clearance rate was shown during the dawn period (5–8 a.m.) compared to the night (1–4 a.m.) [32]. Therefore, since insulin clearance rate—a potential characteristic of the dawn phenomenon—seems to rise physiologically in the morning, an additional stimulation by a CHO-rich breakfast may not be beneficial for the development of body composition, and may be specifically disadvantageous throughout puberty, when insulin resistance is known to be elevated [33].

In the context of circadianity, social jetlag must also be considered. The concept of social jetlag describes a misalignment of biological and social time [34]. Due to the natural chronotype delay during childhood and puberty [35] and an increasing sleep pressure as soon as children enter social institutions [36], social jetlag occurs already in young children [37] and increases until the end of adolescence [34]. The thereby disrupted circadian rhythm may interrelate with macronutrient intake and contribute to an adverse development of body composition, as was already shown for obesity development in adults [34]. Interestingly, data of 305 adolescents revealed that 20% of the association between habitual sleep variability and visceral fat was explained by CHO intake [38]. Additionally, the sleep duration of 767 Danish children aged 8–11 years correlated negatively with higher consumption of added sugar and sugar-sweetened beverages [39]. Therefore, a changing sleep pattern potentially induced by social jetlag may result in higher CHO intake—possibly mainly in the morning, when CHO intake was shown to be highest (Table 2).

Besides the morning, the evening is also discussed as potentially relevant for body composition. Chronobiological studies in adults indicate the already mentioned reduction in insulin sensitivity over the day [26], as well as a reduction in diet-induced thermogenesis [40] and an increasing level of hunger [8], accompanied by a reduced ability to compensate for higher evening energy intakes [41]. Interestingly, in our longitudinal analysis, evening macronutrient intakes did not emerge as relevant exposures for subsequent body composition. 

Moreover, neither morning nor evening energy intake in childhood was associated with body composition in early adolescence. This may in part be attributable to a better ability of children to compensate for higher day-time specific energy intakes at subsequent meals when compared to adults [41,42,43]. Alternatively, we may have been unable to discern existing associations due to the fact that one or two 3-day dietary records may not be sufficient to characterize habitual energy intake.

Results of this study must be considered in the light of several limitations. First, FMI and FFMI were estimated from skinfold thicknesses, which have been criticized for being susceptible to measurement error. However, measurements were performed by trained and quality-monitored study personnel, which resulted in comparably low intra- and inter-observer differences [44]. Moreover, percent body fat estimated from skinfold measurements using the equation proposed by Slaughter [12] was shown to be comparable to, for example, body fat estimation by dual-energy X-ray absorptiometry [45]. Given that the variance in FMI explained by morning fat and CHO intake is smaller than the measurement error, it could be argued that our results reflect chance or bias rather than “true effects”. However, our findings emerged from a two-stage statistical approach (exploratory and in-depth analyses) and remained largely unaffected by adjustment for potential confounders. Nonetheless, confounding by unmeasured covariates remains a possibility. Second, the DONALD population is characterized by a high socio-economic status [9]; i.e., the extremes in nutritional behavior may be under-represented. However, due to the relatively homogeneous sample, our results may be less vulnerable to residual confounding. The overall strengths of the study are the closely-spaced data, including day-time specific nutritional data for every age during childhood. Due to the early recruitment of the study participants and the annually repeated data collection, 3-day weighed dietary records are documented by participants accustomed to the procedure. Moreover, data on several important potential confounders (early-life characteristics, anthropometric, familial, and socio-economic factors) were available.

## 5. Conclusions

Our study suggests that a habitual low fat, high CHO intake during the morning among primary school children may contribute to an increase of their FMI in early adolescence. If confirmed by other studies, adherence to current dietary recommendations favoring a low-fat, high-CHO breakfast may fuel rather than abate the current obesity epidemic among children and adolescents. Further studies are needed to address the potential day-time specific underlying mechanisms.

## Figures and Tables

**Table 1:** Early life, pubertal, familial-, and socio-economic characteristics, as well as data on the outcome body composition (explorative analysis sample, *N* = 372).

Variable	Explorative Analysis Sample
Sex (♀ *n* (%))	182 (48.9)
**Early life factors**	
Birth year	1992 (1987; 1996)
Appropriate for gestational age (*n* (%))	285 (76.6)
Fully breastfed (*n* (%) ≥ 4 months) ^1^	223 (60.1)
**Puberty marker**	
Age at takeoff (ATO, years)	9.7 (8.7; 10.5)
**Socio-economic status**	
Maternal overweight, ≥25 kg/m^2^, (*n* (%)) ^1^	110 (29.7)
Maternal educational status, ≥12 years of schooling, (*n* (%))	223 (60.0)
Smoking in the household (*n* (%))	86 (23.1)
**Body composition at age 10/11 years (Outcome) ^2^**	
BMI (kg/m^2^)	17.6 (16.1; 19.5)
♀	17.5 (16.0; 19.6)
♂	17.7 (16.2; 19.3)
FMI (kg/m^2^)	3.1 (2.3; 4.6)
♀	3.5 (2.5; 4.8)
♂	2.9 (2.0; 4.3)
FFMI (kg/m^2^)	14.3 (13.5; 14.9)
♀	14.0 (13.2; 14.6)
♂	14.5 (13.8; 15.2)
Overweight (*n* (%)) ^3^	60 (16.1)
♀	30 (16.5)
♂	30 (15.8)
Excessive body fatness (*n* (%)) ^4^	78 (20.1)
♀	33 (18.1)
♂	45 (23.7)

Values are shown as *n* (%) for categorized variables and as median (25th; 75th percentile) for continuous variables. BMI—body mass index, FFMI—fat free mass index, FMI—fat mass index, IOTF—international obesity task force, ♀ - girls, ♂ - boys. ^1^ *N* = 371; ^2^ latest available measurement; ^3^ including overweight and obese participants, according to IOTF, Cole, 2000 [19]; ^4^ including overweight and obese participants, according to McCarthy, 2006 [20] with body fat estimation after Slaughter, 1988 [12].

**Table 2:** Dietary characteristics in three potentially critical time periods (explorative analysis sample, *N* = 372).

Exposure	Time Period 1 (Age 2.5 Years–<4.5 Years)	Time Period 2 (Age 4.5 Years–<6.5 Years)	Time Period 3 (Age 6.5 Years–<8.5 Years)	*p* for Trend ^3^
Daily energy intake (MJ)	4.7 (4.3; 5.3)	5.7 (5.2; 6.3)	6.6 (5.9; 7.3)	<0.001
Daily energy intake (kcal)	1132 (1023; 1254)	1364 (1236; 1501)	1564.8 (1409.3; 1735.9)	<0.001
Fat (E% ^1^)	37.0 (33.5; 39.7)	36.2 (33.2; 39.1)	35.3 (32.9; 38.2)	0.002
Carbohydrates (E% ^1^)	50.2 (46.7; 54.2)	51.0 (48.1; 54.4)	52.0 (48.7; 54.7)	0.001
Protein (E% ^1^)	12.8 (11.6; 13.9)	12.6 (11.3; 13.6)	12.6 (11.6; 13.8)	0.055
Energy intake before 11 a.m. (kcal)	347.0 (295.5; 405.2)	379.0 (309.4; 451.2)	438.9 (369.7; 534.0)	<0.001
Energy intake before 11 a.m. (E% ^1^)	30.9 (26.7; 34.8)	27.5 (23.7; 32.9)	28.3 (24.3; 33.1)	<0.001
Fat (E% ^2^)	35.9 (30.9; 40.2)	34.9 (29.9; 39.7)	33.1 (28.9; 37.7)	<0.001
Carbohydrates (E% ^2^)	50.9 (46.3; 57.0)	52.6 (47.6; 58.1)	54.7 (49.6; 58.7)	<0.001
Protein (E% ^2^)	12.8 (11.2; 14.6)	12.3 (10.8; 13.9)	12.4 (10.9; 14.1)	0.013
Energy intake after 6 p.m. (kcal)	259.6 (199.2; 315.3)	334.5 (275.1; 403.5)	417.0 (332.4; 490.2)	<0.001
Energy intake after 6 p.m. (E% ^1^)	22.6 (17.7; 26.9)	24.6 (20.8; 29.0)	26.2 (22.4; 30.2)	<0.001
Fat (E% ^2^)	40.8 (34.8; 46.5)	39.2 (34.0; 45.0)	37.4 (32.6; 41.8)	<0.001
Carbohydrates (E% ^2^)	43.7 (37.4; 51.3)	46.4 (40.1; 52.8)	48.4 (43.2; 54.0)	<0.001
Protein (E% ^2^)	14.2 (12.1; 16.1)	14.0 (12.2; 15.8)	14.0 (12.0; 15.7)	0.361

Values are estimated from one or two 3-day dietary records, shown as median (25th; 75th percentile). ^1^ % of daily energy intake; ^2^ % of energy intake before 11 a.m./after 6 p.m.; ^3^ differences between time periods using Kruskal–Wallis test.

**Table 3:** Relation of fat and carbohydrate (CHO) intake before 11 a.m. during different critical time periods throughout childhood to FMI in early adolescence at age 10/11 years (in-depth analysis sample, *N* = 297).

	Predicted FMI Means in Tertiles of Corresponding Exposures ^1^ (Fat, CHO, ΔFat, ΔCHO)			%Difference T1–T3 ^2^	*p* for Trend ^3^
	Low Intake or Decrease in Intake (T1)	Average Intake or Constant Intake (T2)	High Intake or Increase in Intake (T3)		
**At age 3/4 years**					
Fat (*%E of breakfast* ^4^)					
*Median intake (25th; 75th)*	*28.30 (25.24; 30.70)*	*36.17 (34.65; 37.53)*	*42.26 (40.08; 45;07)*	*+49.3%*	*<0.0001*
Model 1 ^5^	3.21 (2.90–3.55)	3.33 (3.01–3.68)	3.39 (3.06–3.74)	+5.6%	0.49
Model 2 ^6^	3.25 (2.98–3.55)	3.25 (3.09–3.68)	3.30 (3.03–3.60)	+1.5%	0.93
CHO (*%E of breakfast* ^4^)					
*Median intake (25th; 75th)*	*44.16 (40.53; 46.74)*	*50.91 (49.64; 52.14)*	*59.63 (56.95; 62.89)*	*+35.0%*	*<0.0001*
Model 1 ^5^	3.36 (3.04–3.72)	3.39 (3.07–3.75)	3.18 (2.87–3.51)	−5.4%	0.25
Model 2 ^6^	3.26 (2.99–3.56)	3.39 (3.11–3.70)	3.27 (2.99–3.57)	−0.3%	0.80
**At age 7/8 years**					
Fat (*%E of breakfast* ^4^)					
*Median intake (25th; 75th)*	*27.38 (25.07; 29.37)*	*33.20 (32.05; 34.58)*	*40.16 (38.01; 43.40)*	*+46.7%*	*<0.0001*
Model 1 ^5^	3.67 (3.33–4.06)	3.21 (2.90–3.54)	3.07 (2.78–3.39)	−16.4%	0.02
Model 2 ^6^	3.48 (3.28–3.69)	3.30 (3.12–3.50)	3.15 (2.97–3.34)	−9.5%	0.01
CHO (*%E of breakfast* ^4^)					
*Median intake (25th; 75th)*	*45.78 (41.76; 49.30)*	*54.23 (52.02; 56.45)*	*60.25 (58.21; 63.06)*	*+31.6%*	*<0.0001*
Model 1 ^5^	3.14 (2.84–3.47)	3.30 (2.99–3.65)	3.49 (3.16–3.85)	+11.1%	0.14
Model 2 ^6^	3.15 (2.97–3.33)	3.23 (3.05–3.42)	3.56 (3.36–3.77)	+13.0%	0.01
**Change (Δ) between age 3/4 years and age 7/8 years**					
ΔFat (Δ*%E of breakfast* ^4^)					
*Median Δ intake (25th; 75th)*	*−9.02 (−12.54; −6.72)*	*−1.81 (−3.73; 0.17)*	*5.14 (2.67; 8.57)*	*+157.0%*	*<0.0001*
Model 1 ^5^	3.60 (3.26–3.98)	3.25 (2.95–3.59)	3.09 (2.80–3.41)	−14.2%	0.01
Model 2 ^6^	3.55 (3.26–3.87)	3.25 (2.98–3.54)	3.14 (2.88–3.43)	−11.6%	0.02
ΔCHO *(*Δ*%E of breakfast* ^4^*)*					
*Median Δ intake (25th; 75th)*	*−6.02 (−10.41; −2.8)*	*2.53 (−0.38; 3.87)*	*10.28 (8.25; 13.20)*	*−270.8%*	*<0.0001*
Model 1 ^5^	3.13 (2.83–3.46)	3.22 (2.92–3.56)	3.59 (3.25–3.97)	+14.7%	0.02
Model 2 ^6^	3.24 (2.97–3.54)	3.20 (2.93–3.49)	3.49 (3.20–3.81)	+7.7%	0.05

CHO—carbohydrates, FMI—fat mass index, - %E—energy percent; Δ, Change in intake between the age of 3/4 years and 7/8 years (Δ = 7/8 years–3/4 years); ^1^ Model-values are least square means (95% confidence intervals) of the FMI; ^2^ % difference between median intakes or predicted FMI means in tertile 1 and tertile 3; ^3^ *p*-values for differences in median intake or Δ intake are based on Kruskal–Wallis test; *p*-values for model 1 and model 2 are based on linear regression analyses (fat, CHO, Δ fat, Δ CHO as continuous exposure variables); ^4^ Residuals used in linear regression models; ^5^ Model 1 (crude model) adjusted for age at take-off (ATO); ^6^ Model 2 additionally adjusted for baseline FMI; no other covariates emerged as relevant.

## Supplementary Materials

Tables S1–S3 are available online at http://www.mdpi.com/2072-6643/8/11/716/s1. They provide data on the in-depth analysis sample (*N* = 297). Table S1. Early life, pubertal, familial-, and socio-economic characteristics, as well as data on the outcome body composition (in-depth analysis sample, *N* = 297), Table S2. Dietary characteristics for time periods identified as critical (in-depth analysis sample, *N* = 297), Table S3. Relation of morning fat and morning carbohydrate (CHO) intake during different critical time periods throughout childhood to FFMI in early adolescence at age 10/11 years (in-depth analysis sample, *N* = 297).
