# Supplementary material for: Relevance of Morning and Evening Energy and Macronutrient Intake during Childhood for Body Composition in Early Adolescence
Source: Nutrients. 2016 Nov 10;8(11):716. doi: 10.3390/nu8110716 (PMC5133102; doi:10.3390/nu8110716)
Supplement: Supplementary file 1 [file nutrients-08-00716-s001.docx]

Supplementary Materials: Relevance of Morning and Evening Energy and Macronutrient Intake during Childhood for Body Composition in Early Adolescence

Tanja Diederichs, Sarah Roßbach, Christian Herder, Ute Alexy and Anette E. Buyken

The supplemental information provides data on the in-depth analysis sample (*N* = 297).

**Table S1.** Early life, pubertal, familial-, and socio-economic characteristics, as well as data on the outcome body composition (in-depth analysis sample, *N* = 297).

| **Variable** | **In-Depth Analysis Sample** |
| --- | --- |
| Gender (♀ *n* (%)) | 151 (50.8) |
| **Early life factors** |  |
| Birth year | 1992 (1988; 1996) |
| Appropriate for gestational age (*n* (%)) | 227 (76.4) |
| Fully breastfed (*n* (%) ≥ 4 months) | 185 (62.3) |
| **Puberty marker** |  |
| Age at takeoff (ATO, years) | 9.6 (8.7; 10.5) |
| **Socio-economic status** |  |
| Maternal overweight, ≥25 kg/m^2^, (*n* (%)) | 87 (29.3) |
| Maternal educational status, ≥12 years of schooling, (*n* (%)) | 182 (61.3) |
| Smoking in the household (*n* (%)) | 67 (22.6) |
| **Body composition at age 10/11 years (Outcome) ^1^** |  |
| BMI (kg/m^2^) | 17.6 (15.9; 19.5) |
| ♀ | 17.4 (15.8; 19.6) |
| ♂ | 17.8 (16.1; 19.2) |
| FMI (kg/m^2^) | 3.1 (2.3; 4.7) |
| ♀ | 3.4 (2.5; 5.2) |
| ♂ | 2.9 (2.0; 4.3) |
| FFMI (kg/m^2^) | 14.3 (13.4; 14.9) |
| ♀ | 13.9 (13.2; 14.6) |
| ♂ | 14.5 (13.8; 15.1) |
| Overweight (*n* (%)) ^2^ | 48 (16.2) |
| ♀ | 25 (16.6) |
| ♂ | 23 (15.8) |
| Excessive body fatness (*n* (%)) ^3^ | 63 (21.2) |
| ♀ | 29 (19.2) |
| ♂ | 34 (23.3) |

Values are shown as *n* (%) for categorized variables and as median (25th; 75th percentile) for continuous variables. BMI—body mass index, FFMI—fat free mass index, FMI—fat mass index, IOTF—international obesity task force, ♀ - girls, ♂ - boys. ^1^ latest available measurement; ^2^ including overweight and obese participants, according to IOTF, Cole, 2000; ^3^ including overweight and obese participants, according to McCarthy, 2006, with body fat estimation after Slaughter, 1988.

**Table S2.** Dietary characteristics for time periods identified as critical (in-depth analysis sample, *N* = 297).

| **Exposure** | **TIME PERIOD 1  (Age 2.5 Years–<4.5 Years)** | **TIME PERIOD 3  (Age 6.5 Years–<8.5 Years)** | ***p* for Trend ^3^** |
| --- | --- | --- | --- |
| Daily energy intake (MJ) | 4.8 (4.3; 5.2) | 6.5 (5.9; 7.2) | <0.001 |
| Daily energy intake (kcal) | 1135 (1037; 1254) | 782.5 (747.0; 823.8) | <0.001 |
| Fat (E% ^1^) | 37.0 (33.6; 39.7) | 35.5 (32.9; 38.2) | 0.003 |
| Carbohydrates (E% ^1^) | 50.2 (46.7; 54.0) | 51.7 (48.5; 54.5) | 0.004 |
| Protein (E% ^1^) | 12.8 (11.5; 13.8) | 12.6 (11.7; 13.8) | 0.731 |
| Energy intake before 11 a.m. | 349.8 (301,2; 403.9) | 446.9 (369.5; 525.2) | <0.001 |
| Energy intake before 11 a.m. (E% ^1^) | 31.1 (26.9; 34.8) | 28.3 (24.4; 32.8) | <0.001 |
| Fat (E% ^2^) | 36.2 (30.7; 40.1) | 33.2 (29.2; 38.0) | <0.001 |
| Carbohydrates (E% ^2^) | 50.9 (46.6; 56.9) | 54.3 (49.3; 58.5) | <0.001 |
| Protein (E% ^2^) | 12.8 (11.2; 14.6) | 12.5 (11.0; 14.2) | 0.159 |
| Energy intake after 6 p.m. | 261.2 (204.4; 309.0) | 412.4 (334.6; 489.3) | <0.001 |
| Energy intake after 6 p.m. (E% ^1^) | 22.6 (18.1; 26.6) | 26.2 (22.3; 30.3) | <0.001 |
| Fat (E% ^2^) | 40.8 (35.1; 46.0) | 37.7 (33.2; 42.2) | <0.001 |
| Carbohydrates (E% ^2^) | 43.6 (37.9; 50.6) | 48.1 (42.7; 53.5) | <0.001 |
| Protein (E% ^2^) | 14.2 (12.1; 16.2) | 14.0 (12.1; 15.8) | 0.536 |

Values are shown for time periods 1 and 3, which were identified as relevant for the in-depth analysis (see methods, statistical analysis). Values are estimated from two 3-day dietary records, shown as median (25th; 75th percentile). ^1^ % of daily energy intake; ^2^ % of energy intake before 11 a.m./after 6 p.m.; ^3^ differences between time periods using Wilcoxon’s rank-sum test.

**Table S3.** Relation of morning fat and morning carbohydrate (CHO) intake during different critical time periods throughout childhood to FFMI in early adolescence at age 10/11 years (in-depth analysis sample, *N* = 297).

|  | **Predicted FFMI Means in Tertiles of Corresponding Exposures ^1^ (Fat, CHO, ΔFat, ΔCHO)** | | | **% Difference T1–T3 ^2^** | ***p* for Trend ^3^** |
| --- | --- | --- | --- | --- | --- |
|  | **Low Intake or Decrease in Intake (T1)** | **Average Intake or Constant Intake (T2)** | **High Intake or Increase in Intake (T3)** |  |  |
| **At age 3/4 years** | | | | | |
| Fat (*%E of breakfast* ^4^) |  |  |  |  |  |
| *Median intake (25th; 75th)* | *28.30 (25.24; 30.70)* | *36.17 (34.65; 37.53)* | *42.26 (40.08; 45;07)* | *+49.3%* | *<0.0001* |
| Model 1 ^5^ | 14.29 (14.07–14.51) | 14.37 (14.15–14.59) | 14.11 (13.89–14.33) | −1.3% | 0.59 |
| Model 2 ^6^ | 14.29 (14.12–14.45) | 14.23 (14.07–14.40) | 14.24 (14.08–14.41) | −0.4% | 0.91 |
| CHO (*%E of breakfast* ^4^) |  |  |  |  |  |
| Median intake *(25th; 75th)* | *44.16 (40.53; 46.74)* | *50.91 (49.64; 52.14)* | *59.63 (56.95; 62.89)* | *+35.0%* | *<0.0001* |
| Model 1 ^5^ | 14.22 (14.00–14.44) | 14.24 (14.02–14.47) | 14.30 (14.08–14.52) | +0.6% | 0.98 |
| Model 2 ^6^ | 14.29 (14.13–14.46) | 14.15 (13.98–14.31) | 14.33 (14.16–14.49) | +0.3% | 0.85 |
| **At age 7/8 years** | | | | | |
| Fat (*%E of breakfast* ^4^) |  |  |  |  |  |
| *Median intake (25th; 75th)* | *27.38 (25.07; 29.37)* | *33.20 (32.05; 34.58)* | *40.16 (38.01; 43.40)* | *+46.7%* | *<0.0001* |
| Model 1 ^5^ | 14.44 (14.22–14.66) | 14.17 (13.95–14.39) | 14.16 (13.94–14.37) | −1.9% | 0.03 |
| Model 2 ^6^ | 14.23 (14.10–14.37) | 14.26 (14.13–14.39) | 14.27 (14.14–14.40) | +0.3% | 0.61 |
| CHO (*%E of breakfast* ^4^) |  |  |  |  |  |
| *Median intake (25^th^; 75^th^)* | *45.78 (41.76 49.30)* | *54.23 (52.02; 56.45)* | *60.25 (58.21; 63.06)* | *+31.6%* | *<0.0001* |
| Model 1 ^5^ | 14.20 (13.98–14.42) | 14.23 (14.01–14.46) | 14.33 (14.11–14.55) | +0.9% | 0.15 |
| Model 2 ^6^ | 14.28 (14.15–14.41) | 14.23 (14.10–14.36) | 14.26 (14.13–14.39) | −0.1% | 0.60 |
| **Change (**Δ**) between age 3/4 years and age 7/8 years** | | | | | |
| ΔFat (Δ*%E of breakfast* ^4^) |  |  |  |  |  |
| *Median Δ intake (25th; 75th)* | *−9.02 (−12.54; −6.72)* | *−1.81 (−3.73; 0.17)* | *5.14 (2.67; 8.57)* | *+157.0%* | *<0.0001* |
| Model 1 ^5^ | 14.24 (14.02–14.46) | 14.33 (14.11–14.55) | 14.19 (13.97–14.41) | −0.4% | 0.18 |
| Model 2 ^6^ | 14.23 (14.07–14.40) | 14.35 (14.19–14.51) | 14.18 (14.02–14.34) | −0.4% | 0.42 |
| ΔCHO (Δ*%E of breakfast* ^4^) | | | |  |  |
| *Median Δ intake (25th; 75th)* | *−6.02 (−10.41; −2.8)* | *2.53 (−0.38; 3.87)* | *10.28 (8.25; 13.20)* | *−270.8%* | *<0.0001* |
| Model 1 ^5^ | 14.28 (14.06–14.51) | 14.25 (14.03–14.47) | 14.23 (14.01–14.45) | −0.4% | 0.18 |
| Model 2 ^6^ | 14.32 (14.15–14.48) | 14.25 (14.08–14.41) | 14.20 (14.03–14.36) | −0.8% | 0.62 |

CHO—carbohydrates, FFMI—fat free mass index, y—years, %E—energy percent; Δ—Change in intake between the age of 3/4 years and 7/8 years (Δ = 7/8 years–3/4 years). ^1^ Model-values are least square means (95% confidence intervals) of the FFMI; ^2^ % difference between median intake or predicted FFMI mean in tertile 1 and tertile 3; ^3^ *p*-values for differences in median intake or Δ intake are based on Kruskal–Wallis-test; *p*-values for model 1 and model 2 are based on linear multiple regression analyses (fat, CHO, Δfat, ΔCHO as continuous exposure variables), with *p* < 0.05 defined as significant;
^4^ Residuals used in linear regression model; ^5^ Model 1 (crude model) adjusted for age at take-off (ATO); ^6^ Model 2 additionally adjusted for baseline FFMI; no other covariates emerged as relevant.
